# Lower Circulating Leptin Levels Are Related to Non-Alcoholic Fatty Liver Disease in Children With Obesity

**DOI:** 10.3389/fendo.2022.881982

**Published:** 2022-05-23

**Authors:** Stephanie Brandt, Julia von Schnurbein, Christian Denzer, Wolfgang Kratzer, Martin Wabitsch

**Affiliations:** ^1^ Center for Rare Endocrine Diseases, Division of Paediatric Endocrinology and Diabetes, Department of Paediatrics and Adolescent Medicine, University Medical Center Ulm, Ulm, Germany; ^2^ Department of Internal Medicine I, University Hospital Ulm, Ulm, Germany

**Keywords:** obesity, children, NAFLD, leptin, partial leptin deficiency

## Abstract

**Background:**

While for individuals with obesity an association between hyperleptinemia and an increased risk of non-alcoholic fatty liver disease (NAFLD) is assumed, a leptin deficiency is also related to the development of NAFLD early in life in *ob/ob* mice, in patients with leptin deficiency due to biallelic likely pathogenic variants in the leptin gene, and in patients with lipodystrophy.

**Objectives:**

To investigate the association of circulating leptin levels in pre-pubertal children with obesity and steatosis hepatis.

**Methods:**

The cross-sectional study consisted data of n=97 (n_male_=76) pre-pubertal children (11.8 ± 1.5 years) with obesity (BMIz: 2.4 ± 0.4). Fasting concentrations of cardiometabolic parameters were measured: insulin, c-peptide, glucose, triglyceride, cholesterol, HDL, LDL, AST, ALT, GGT, leptin. Steatosis hepatis was diagnosed by an ultrasound examination (mild, moderate or severe). Patients were categorized into two groups: low z-score of circulating leptin levels (≤25th percentile) vs. normal z-score of circulating leptin levels.

**Results:**

One-third of the children with obesity were diagnosed with steatosis hepatis (I°: 63.6%, II°/III°: 36.4%). Children with steatosis hepatis had significantly lower z-scores of circulating leptin levels compared to children with an unremarkable liver ultrasonography (-2.1 ± 0.8 vs. -0.7 ± 0.6). Z-scores of circulating leptin levels correlate negatively with degree of steatosis hepatis. Children with low z-scores of circulating leptin levels had significantly higher triglyceride, fasting insulin and c-peptide levels compared to children with normal z-scores of circulating leptin levels.

**Conclusion:**

Prepubertal children with NAFLD and obesity and partial leptin deficiency might be defined as a clinical subgroup.

## Introduction

Children with obesity are at risk for numerous health problems, including nonalcoholic fatty liver disease (NAFLD). The estimated prevalence of NAFLD is 3–10% of children worldwide, and ranged between 26.4% and 34.2% in children with obesity in epidemiological studies (1–3). Studies have demonstrated that there is a high risk for a progression of pediatric NAFLD to end-stage liver disease in adulthood. This emphasizes the importance of research in effective prevention and intervention strategies. As no pharmacological agents for the treatment of pediatric NAFLD exist, the cornerstone therapeutic strategy is lifestyle intervention. A recently published review demonstrated, that the positive effects of a lifestyle intervention on liver-associated parameters are weak and are strongly associated with body weight loss (4). Since lifestyle interventions are largely unsuccessful in achieving and maintaining clinically meaningful weight loss (5, 6), the search for new pharmacological interventions in adults and children with NAFLD is on focus. One possible approach would be to further investigate the role of the adipokine leptin in relation to the development of NAFLD (7, 8). Hyperleptinemia is thought to be associated with an increased risk of NAFLD in children, adolescents, and adults with obesity (9). In contrast, there are observations from *ob/ob* mice and from patients with leptin deficiency due to biallelic likely pathogenic variants in the leptin gene, in which liver steatosis has also been described (10, 11). Metreleptin substitution in patients with leptin deficiency results in a reduction of liver fat content prior to any detectable weight reduction (11). The treatment of obesity by Metreleptin was already studied in the 2000s. In several intervention studies in subjects with obesity, high doses of Metreleptin failed to show an effect on body weight loss (12). However, there was a subgroup of patients with low circulating leptin levels at baseline who benefited from the intervention with Metreleptin in terms of greater body weight loss (13). In a recently published open-label therapy intervention study, Akinci et al. investigated, whether Metreleptin administration for 12 months has an impact on the global NASH score in adults with obesity and with relative leptin deficiency (individual leptin level <25th percentile for leptin levels related to age and BMI of a reference group). Akinici et al. described that there was significant weight loss and significant reduction in adults with obesity and with relative leptin deficiency in the global NASH score after Metreleptin administration for 12 months (14). A state of relative leptin deficiency could be defined by low circulating leptin levels relative to BMI. Since there is no uniform, widely accepted definition of “relative leptin deficiency” in patients with obesity, the question arises how to identify patients with relatively low leptin levels. During childhood and adolescence, leptin levels depend primarily on sex and BMI (fat mass) and rise with progression of pubertal development. Blum et al. developed a formula for the calculation of the z-score for circulating leptin levels in childhood and adolescence, in which pubertal stage, sex and BMI are considered (15). In the present study, we hypothesize that there is an association between reduced z-scores of circulating leptin levels and diagnosis of steatosis hepatis in pre-pubertal children with obesity.

## Methods

### Study Population

The study population consists data of n=97 children with obesity, who were admitted to an inpatient rehabilitation clinic (Murnau, Germany) to participate in a weight loss program. The clinical examination at admission to the clinic included anthropometric measurements, ascertainment of Tanner stage (16, 17), withdrawal of a fasting blood sample, a liver ultrasonography and a questionnaire about medications. Study sample inclusion criteria were prepubertal age and no intake of medications potentially affecting insulin, glucose, aminotransferase or cholesterol levels.

Written informed consent from parents and written assent from children were obtained. The institutional ethical review board of the university of Ulm approved all study proceedings.

### Anthropometrics

Weight and height were measured to the nearest 0.1 kg and 0.1 cm. Individual BMI value was calculated (weight divided by the square of height; kg/m^2^). Individual BMI values were converted into standard deviation scores (BMI-SDS) using the LMS method (18).

### Ultrasound Examinations

An ultrasound examination was performed by a single examiner experienced in sonography (4000 ultrasound examinations per year and accredited instructor of the German Society for Ultrasound in Medicine (DEGUM)). The examinations were performed with the ultrasound device Versa Plus from Siemens, Erlangen, Germany. For imaging of liver, gallbladder, and aorta, a 3.5 MHz convex transducer was used. The ultrasound gel was the product “Ultrasound contact gel” from the company Wasserfuhr (Caesar and Loretz, Hilden, Germany). In order to create the best possible conditions for sonographic assessment of the upper abdomen, patients were called in after a fasting period of at least five hours. Fasting state was defined as: no food, no drinks, no chewing gum, and no cigarettes. The diagnosis of steatosis hepatis was made by one examiner based on a comparison of the hepatic and renal parenchyma, taking into consideration the dorsal attenuation of the diaphragm and ability to assess the liver vessels. According to established diagnostic criteria (19, 20), the degree of fatty infiltration of the liver was classified as “mild” (I°): liver parenchyma more echoic, no dorsal sound reduction; “moderate” (II°): additional dorsal sound reduction, diaphragm still presentable; “severe” (III°): dorsal sound reduction, diaphragm can no longer be visualized. Due to the small number of cases in group III° (severe), these subjects were combined with those in group II° (moderate or severe) for the statistical analysis.

### Biochemical Analyses- Fasting Blood Sample

Fasting blood samples were obtained after an overnight fast. Plasma glucose was measured with the GOD-PAP, serum cholesterol concentrations were measured with the CHOD-PAP, triglyceride with the GPO-PAP method on a LP 700 system (Dr. Lange, GmbH, Berlin, Germany). HDL concentrations were measured by the method precipitation with dextran sulfate/magnesium chloride. LDL concentrations were measured by precipitation of LDL with polyanions and calculation of LDL cholesterol from total cholesterol and cholesterol in the precipitation supernatant. Fasting plasma insulin and C-peptide concentrations were measured using immunoassays (Insulin RIA 100; Pharmacia & Upjohn, Kalamazoo, MI; and C-PEPTID EIA-1293, DRG, Instruments, Marburg, Germany). Fasting AST Aspartat-Aminotransferase (AST), Alanin-Aminotransferase (ALT) and Glutamat-Pyruvat-Transaminase (GGT) (U/l) were measured using the Dimension RxL system (Dade Behring, Eschborn, Germany) applying standard methods (DuPont, Billerica, MA). Adiponectin was measured using an enzyme immunoassay (Quantikine1; R&D Systems, Minneapolis, MN) with an intraassay coefficient of variation of 7.2%). Leptin levels were quantified using enzyme-linked immunosorbent assay (Mediagnost, Reutlingen, Germany). To investigate the research question, we calculated the sex, Tanner stage and BMI dependent z-score for circulating leptin levels using the formula published by Blum et al. (15). We used the 25th internal percentile of the z-score of circulating leptin levels to define two groups of children: (A) children with low z-scores of circulating leptin levels: z-score for circulating leptin levels (≤25th internal percentile), (B) children with normal z-scores of circulating leptin levels (>25th internal percentile).

### Statistical Analyses

Data are presented as means, standard error of the mean (SE), median and interquartile range (IQR) for continuous variates and as percentages for categorical variates. Box plots are composed of five horizontal lines that display the10th, 25th, 50th, 75th and 90th percentiles of available. To test for group differences the Student´s t-test was performed. Kruskal–Wallis tests and analyses of covariance were used to compare means of z-scores of circulating leptin levels across patient groups with no, mild, moderate or severe liver steatosis. All tests were performed with the Statistical Analyses System version 9.4 (Statistical Analyses System Institute Inc., Cary, North Carolina, USA). Statistical significance was inferred at two-tailed p<0.05.

## Results

### Prevalence of NAFLD

The basic characteristics of the cohort of pre-pubertal children with obesity in total and separated for males and females are shown in **Table 1**. The majority of children in the cohort were male (male: 78.4 vs. female: 21.7%). The BMI z-score was comparable between males and females. One-third of the pre-pubertal children with obesity were diagnosed with hepatic steatosis by ultrasound. The majority of the pre-pubertal children with obesity were affected by mild fatty infiltration of the liver (steatosis hepatis I°: 63.6%) and 36.4% were affected by moderate or severe liver steatosis (steatosis hepatis II° or III°). The percentage of pre-pubertal children with obesity and with steatosis hepatis was higher in males than in females (male: 38.2 vs. female: 19.1%).

**Table 1:** Description of the anthropometric and metabolic parameters (adipokine concentration, concentration of liver enzymes, insulin and lipid metabolism), as well as of the distribution of pre-pubertal children with obesity and with steatosis hepatis in the whole cohort (n=97), as well as separated for males (n=76; 78.4% of cohort) and females (n=21; 21.6% of cohort).

	Whole cohort				Male				Female		
	mean±STD	median	IQR		mean±STD	median	IQR		mean±STD	median	IQR
Age [years]	11.8±1.5	11.8	10.7–12.8		12.0±1.3	12.1	11.0–12.9		11.0±1.8	10.8	9.8–12.0
**Anthropometrics**											
BMI SDS	2.4±0.4	2.4	2.1–2.7		2.4±0.4	2.4	2.1–2.7		2.4±0.5	2.4	2.2–2.7
BMI [kg/m^2^]	29.4±4.0	29.0	26.8–32.0		29.7±3.9	29.1	27.0–32.2		28.2±3.9	27.8	26.1–29.7
**Adipokines**											
Adiponectin [µg/ml]	6.4±2.7	5.8	4.4–8.2		6.3±2.7	5.7	4.4–7.8		6.9±2.9	5.9	4.4–9.4
Leptin [ng/ml]	27.1±13.8	23.7	17.5–32.9		27.7±13.0	24.9	18.6–33.1		25.1±16.4	20.9	14.7–27.7
Z-score circulating leptin level	-1.2±1.6	-1.2	-2.2–-0.1		-1.1±1.6	-1.1	-2.2–0.0		-1.7±1.4	-1.8	-2.8–-0.8
**Liver enzymes**											
GGT [U/l]	14.8±11.0	12.7	10.8–15.1		15.7±12.2	12.9	11.3–16.7		11.6±3.7	10.9	8.8–13.7
AST [U/l]	15.4±5.9	14.3	12.2–16.1		15.8±6.5	14.1	12.1–17.5		13.9±2.6	14.6	12.8–15.7
ALT [U/l]	15.3±8.6	12.6	10.7–17.2		15.8±9.2	12.3	10.8–17.3		13.6±5.5	13.0	10.4–14.6
**Insulin metabolism**											
Fasting glucose [mg/dl]	83.4±8.5	82.4	78.4–88.0		84.4±7.3	83.4	79.7–88.1		79.7±11.3	79.7	73.0–83.8
Fasting insulin [mU/l]	14.7±11.0	11.4	8.8–16.1		15.7±11.8	11.9	9.2–17.4		11.0±7.0	9.1	5.4–14.4
Fasting c-peptide [µg/l]	1.7±0.9	1.5	1.1–1.9		1.8±0.9	1.5	1.2–1.9		1.4±0.7	1.2	0.8–1.8
**Lipid metabolism**											
HDL [mg/dl]	47.7±9.2	46.7	41.5–52.3		48.3±9.5	47.0	41.3–54.5		45.5±7.6	45.2	41.5–47.6
LDL [mg/dl]	129.0±33.9	127.0	100.0–153.0		130.3±34.6	128.0	100.0–154.5		124.4±31.3	123.0	100.0–141.0
TG [mg/dl]	103.1±63.8	85.7	63.0–120.0		108.5±67.7	91.5	66.6–123.5		83.5±42.8	74.4	61.9–85.7
Cholesterol [mg/dl]	198.3±33.9	197.0	176.6–220.6		200.8±34.0	198.5	177.5–225.0		189.4±32.7	186.1	171.0–206.0
**Steatosis hepatis [% (n)]**											
No	66.0 (64)				61.8 (47)				81.0 (17)		
Yes	34.0 (33)				38.2 (29)				19.0 (4)		
**Steatosis hepatis [% (n)]**											
I° (mild)	63.6 (21)				58.6 (17)				100 (4)		
II° or III° (moderate or severe)	36.4 (12)				41.4 (12)				0 (0)		

Parameter values are presented as means±standard deviation (STD), medians and interquartile ranges (IQR).

### Pre-Pubertal Children With Obesity and With NAFLD Had Lower z-Scores of Circulating Leptin Levels Than Those Without NAFLD

**Figure 1:**
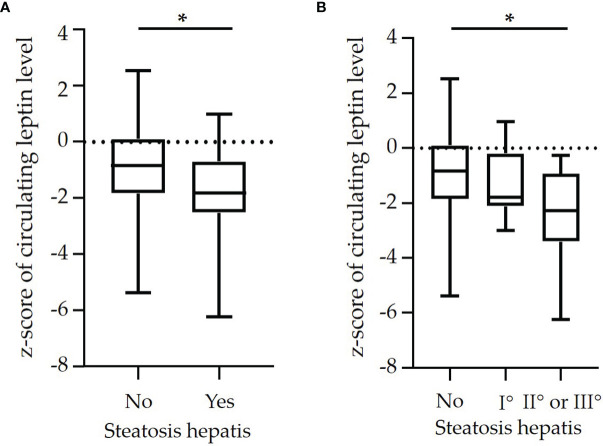
Box plots of z-scores of circulating leptin levels in dependence on **(A)** diagnosis of steatosis hepatis [yes vs. no (n=64 vs. n=33)] and in dependence on **(B)** degree of steatosis hepatis [no steatosis hepatis (n=64] vs. mild (I°: n=21) vs. moderate or severe [II° or III°: n=12)] in a cohort of n=97 pre-pubertal children with obesity. (*p<0.05).

Children with steatosis hepatis had significantly lower z-scores of circulating leptin levels compared to children with an unremarkable liver ultrasonography finding (**Figure 1A**). The z-scores of circulating leptin levels declined with increasing severity of steatosis hepatis (**Figure 1B**).

### Higher Proportion of Children With NAFLD in the Group of Children With Low z-Scores of Circulating Leptin Levels Than in the Control Group

**Figure 2:**
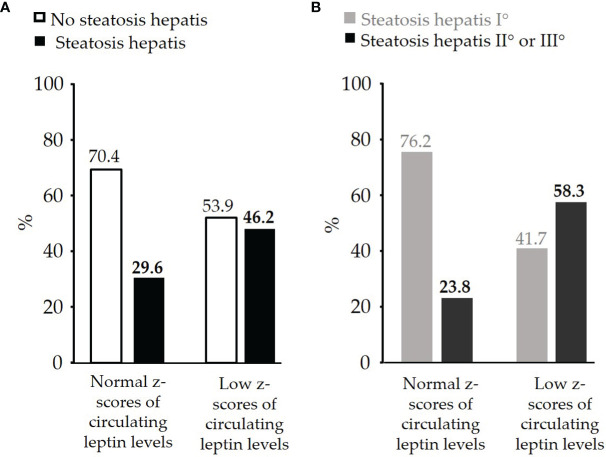
**(A)** Comparison of percentage of pre-pubertal children with obesity and with or without diagnosed steatosis hepatis between the group of children with normal z-scores of circulating leptin levels (n=71) and the group of children with low z-scores of circulating leptin levels (n=26), **(B)** Comparison of percentage of pre-pubertal children with obesity and with steatosis hepatis I° (mild: n=21) or steatosis hepatis II° or III° (moderate or severe: n=12) between the group of children with normal z-scores of circulating leptin levels and the group of children with low z-scores of circulating leptin levels.

The percentage of children affected by steatosis hepatis was higher in the group of children with low z-scores of circulating leptin levels than in children with normal z-scores of circulating leptin levels (46.2% vs. 29.6%) (**Figure 2A**). Among the children with steatosis hepatis, those children with low z-scores of circulating leptin levels had more often a moderate or even severe degree of steatosis hepatis compared to children with normal z-scores of circulating leptin levels (58.3% vs. 23.8%) (**Figure 2B**).

### Children With Low z-Score of Circulating Leptin Levels Had Higher Triglyceride, Fasting Insulin and c-Peptide Levels Compared to Children of the Control Group

Pre-pubertal children with obesity and with low z-scores of circulating leptin levels show significantly higher liver enzyme levels of AST (17.3 ± 6.8 vs. 14.7 ± 5.5 U/l, p<0.05) and ALT (18.4 ± 9.1 vs. 14.2 ± 8.1 U/l, p<0.05), higher fasting insulin (16.8 ± 13.4 vs. 13.9 ± 10.0 mU/l, p<0.05) and c-peptide levels (2.0 ± 1.1 vs. 1.6 ± 0.7 µg/l, p<0.05), higher triglyceride (114.9 ± 76.7 vs. 98.8 ± 58.3, p<0.05), higher total cholesterol and lower HDL cholesterol levels (44.7 ± 8.0 vs. 48.8 ± 9.4 mg/dl, p<0.05) compared to children with normal z-scores for circulating leptin levels (**Table 2**). Levels of adiponectin, fasting glucose and LDL cholesterol were not significantly different between the two groups of children. BMI z-scores (2.8 ± 0.2 vs. 2.2 ± 0.4, p<0.05) were higher in the group of children with low z-scores of circulating leptin levels than in children with normal z-scores for circulating leptin levels.

**Table 2:** Comparison of anthropometric and metabolic parameters (adipokine, liver enzymes, insulin and lipid metabolism) as well as of the percentage of pre-pubertal children with obesity and with diagnosed steatosis hepatis between the group of children with normal z-scores of circulating leptin levels (n=71) and the group of children with low z-scores of circulating leptin levels (n=26).

	Normal z-scores of circulating leptin levels [n=71]				Low z-scores of circulating leptin levels [n=26]			
	mean±STD	median	IQR		mean±STD	median	IQR	p-value
Age [years]	11.6±1.5	11.6	10.7–12.7		12.2±1.4	12.6	10.9–13.1	>0.05
**Anthropometrics**								
BMI z-score	2.2±0.4	2.2	2.0–2.5		2.8±0.2	2.8	2.7–3.0	<0.05
BMI [kg/m^2^]	27.6±2.7	27.9	25.8–29.5		34.1±3.0	34.3	32.1–35.7	<0.05
**Adipokine**								
Adiponectin [µg/ml]	6.6±2.9	6.4	4.4–9.0		5.8±2.1	5.5	4.1–7.3	>0.05
Leptin [ng/ml]	24.2±11.3	22.0	16.3–30.3		35.1±16.7	31.9	21.5–44.8	<0.05
Z-score circulating leptin level	-0.5±1.1	-0.7	-1.5–0.1		-3.2±1.0	-2.9	-3.6–-2.5	<0.05
**Liver enzymes**								
GGT [U/l]	15.1±12.6	12.7	10.9–14.7		14.0±5.1	12.5	10.6–17.1	>0.05
AST [U/l]	14.7±5.5	13.9	11.9–16.1		17.3±6.8	14.7	14.0–18.7	<0.05
ALT [U/l]	14.2±8.1	12.0	9.6–16.6		18.4±9.1	15.3	12.1–20.4	<0.05
**Insulin metabolism**								
Fasting glucose [mg/dl]	84.7±8.4	83.6	79.8–88.2		79.9±7.7	79.4	76.1–85.0	>0.05
Fasting insulin [mU/l]	13.9±10.0	11.3	8.9–15.6		16.8±13.4	13.4	8.6–20.4	<**0.05**
Fasting c-peptide [µg/l]	1.6±0.7	1.4	1.1–1.8		2.0±1.1	1.6	1.3–2.5	<0.05
**Lipid metabolism**								
HDL [mg/dl]	48.8±9.4	47.6	41.5–52.9		44.7±8.0	44.7	38.1–47.3	<0.05
LDL [mg/dl]	131.6±30.9	131.0	107.0–154.0		121.8±40.6	115.5	89.0–152.0	>0.05
TG [mg/dl]	98.8±58.3	85.7	61.9–119.0		114.9±76.7	82.7	65.4–124.0	<0.05
Cholesterol [mg/dl]	202.2±29.6	198.0	185.7–220.6		187.8±42.5	181.0	150.0–223.0	<0.05
**Steatosis hepatis [% (n)]**								
No	70.4 (50)				53.8 (14)			<0.05
Yes	29.6 (21)				46.2 (12)			
**Steatosis hepatis [% (n)]**								
No	70.4 (50)				53.8 (14)			
I° (mild)	22.2 (16)				19.2 (5)			
II° or III° (moderate or severe)	7.4 (5)				27.0 (7)			<0.05

Parameter values are presented as means±standard deviation (STD), medians and interquartile ranges (IQR).

## Discussion

The aim of this work was to investigate the relationship between z-scores of circulating leptin levels and the prevalence and the degree of steatosis hepatis in a well-characterized cohort of pre-pubertal children with obesity. We observed that pre-pubertal children with obesity and with steatosis hepatis have significantly lower z-scores of circulating leptin levels than children with an unremarkable liver sonographic finding, and z-scores of circulating leptin levels correlate negatively with degree of steatosis hepatis. Furthermore, the percentage of children with all degrees of steatosis hepatis was higher in the group of children with low z-scores of circulating leptin levels compared to children with normal z-scores of circulating leptin levels.

### Pre-Pubertal Children With Obesity and With NAFLD Have Low z-Scores of Circulating Leptin Levels

We have shown that pre-pubertal children with obesity and with NAFLD have significantly lower z-scores of circulating leptin levels than children without NAFLD. Few studies have examined the association between circulating leptin levels and steatosis hepatis in children and adolescents with obesity. In the clinical trial “Exercise Training and Hepatic Metabolism in Overweight/Obese Adolescents (HEPAFIT) with steatosis hepatis” (n=122 adolescents (11-17 years)) leptin levels and CAP (indicator of fat deposition in the liver) as well as HOMA-IR and CAP were positively associated, even after adjustment for age, sex and body fat (21). In another study higher circulating leptin levels in n=72 children and adolescents (9-18 years) correlated with more severe degree of steatosis hepatis, ballooning and NAFLD activity score (NAS) (9). In a further analysis of this cohort, the patients were divided into two groups according to their NAS score: ≥5 vs. ≤4. The group of children and adolescents with a NAS score ≥5 showed significantly higher circulating leptin, TNF-alpha, triglyceride, and γGT levels and higher BMI z-scores than the group of children and adolescents with a NAS score ≤4 (22). Taken together, these studies suggest a positive association between circulating leptin levels and NAFLD and also with degree of NAFLD in children and adolescents with obesity. We would like to point out that all of these studies have a limitation, since the circulating leptin levels were analyzed without the consideration of the factors that influence circulating leptin levels during childhood and adolescents: Tanner stage, sex and BMI. In contrast to the studies described above, we calculated z-scores for circulating leptin levels according to references published by Blum et al. (15) and we showed that pre-pubertal children with obesity and with NAFLD had lower z-scores of circulating leptin levels than children without NAFLD.

We used the 25^th^ internal percentile of z-scores of circulating leptin levels as cut-off to identify pre-pubertal children with obesity and with low z-scores of circulating leptin levels. A similar approach was used by Akinci et al, who defined relative leptin deficiency in adults as circulating leptin levels below the 25th percentile of a BMI and sex-matched American population (14). Akinci et al. hypothesized that there are adults with obesity and with relative leptin deficiency and NAFLD in whom treatment with exogenous Metreleptin administration may improve liver parameters. The assumption, that there are individuals among the population with common obesity who have low circulating leptin levels and the opportunity for leptin to act when its level raised from low (below physiological level) to normal, are strengthened by two studies in adults with obesity treated with Metreleptin (12, 13). While the majority of patients with obesity did not benefit from this treatment, patients with reduced basal leptin levels showed an improved reduction in fat mass (12) or body weight under treatment with Metreleptin (13).

### Role of the Adipokine Leptin in the Development of the NAFLD

We observed that pre-pubertal children with obesity and with low z-scores of circulating leptin levels had significantly higher triglyceride, fasting insulin and fasting c-peptide levels compared to children with normal z-scores of circulating leptin levels. This may at least partially be explained by relative leptin deficiency. We assume that these pre-pubertal children with obesity have too low circulating leptin levels related to their BMI. The physiological role of leptin and the anti-steatotic effect of leptin cannot be fully mediated. Our hypothesis is supported by observations from animal and clinical studies. In leptin-deficient *ob/ob* mice as well as in patients with congenital leptin deficiency, a striking metabolic phenotype including severe degree of steatosis hepatis has been described. Metreleptin substitution in humans with congenital leptin deficiency reverses steatosis hepatis as shown by our group recently (11). Furthermore, a systematic literature review provided evidence for a higher frequency of metabolic abnormalities in LEP *wt/-* than in *wt/wt* subjects, including hypercholesterinemia, hyperinsulinemia, and hypertriglyceridemia. Animal studies demonstrated lower leptin levels in LEP *wt/-* compared to *wt/wt* animals, especially in relation to fat mass (23). Furthermore, in humans with lipodystrophies, a group of disorders that are characterized by a selective deficiency of subcutaneous adipose tissue and low circulating leptin levels, severe forms of NAFLD have been described together with other metabolic complications including insulin resistance and high triglyceride levels. Leptin replacement therapy in lipodystrophic patients leads to an improvement in ectopic lipid disposition and in fatty liver disease (24). Also, in normal weight adults, 5-8% are diagnosed with NAFLD (lean NAFLD) (25). It has been shown that normal weight adults (Caucasian) with NAFLD (lean NAFLD) had significantly lower leptin levels than obese adults with NAFLD, but circulating leptin levels did not differ between lean healthy and lean NAFLD adults (26). But it should be considered that in this study the circulating leptin levels in lean NAFLD and healthy adults were compared without considering the age, BMI, and gender specific effect on circulating leptin levels. The question remains open whether lean NAFLD adults have lower z-scores for circulating leptin levels compared to healthy lean adults.

### Metreleptin – a Treatment Option for NAFLD in Children With Obesity?

As no pharmacological treatment for NAFLD in childhood and adolescence is currently available, the cornerstone of therapeutic strategies for pediatric NAFLD remains lifestyle intervention. A review published in 2020 summed up the results of n=10 RCTs assessing the efficiency of dietary and lifestyle interventions on several NAFLD-related parameters in children and adolescents with imaging or biopsy-proven NAFLD. All interventions described an improvement in liver outcomes in conjunction with weight loss (4). It is known that it is very difficult to achieve clinically relevant and lasting body weight reduction in children and adolescents with obesity or extreme obesity by lifestyle intervention (5, 6). Since the effective treatment of NAFLD by lifestyle modification has no sustainable effect, the search for pharmacological agents is of high relevance. One approach in adults was to study the treatment of NAFLD with Metreleptin. In adult patients with lipodystrophy and NAFLD it has been shown, that treatment with Metreleptin resulted in a significantly reduced liver volume and steatosis (24). In adults with obesity and with relative leptin deficiency, Akinci et al. showed within an open-label therapy intervention study, that the global NASH score was significantly reduced under Metreleptin administration for 12 months (12). We hypothesize that Metreleptin might be a treatment option in children with obesity, NAFLD and with low z-scores of circulating leptin levels with the aim to improve fatty infiltration and hepatic inflammation.

### Limitations

We calculated z-scores for circulating leptin levels, which consider the dependency of circulating leptin levels on sex, Tanner stage and BMI during childhood and adolescence. The reference values were based on a cohort of children and adolescents older than 6 years of age who were primarily normal weight (15). Although the extreme ranges for upper (overweight/obesity) and lower BMI values (underweight) are not present in this reference cohort, we assume that these references can be used to detect discrepancies between given BMI value and circulating leptin levels. Within the presented study, liver ultrasound examination was conducted in pre-pubertal children with obesity at one time point. Liver ultrasound measurement of these patients at the end of lifestyle intervention would be necessary to study if pre-pubertal children with obesity and with low z-scores of circulating leptin levels at baseline showed a stronger improvement in parameters of liver metabolism and liver steatosis under lifestyle intervention than children with normal z-scores of circulating leptin levels.

### Conclusion

In conclusion, we showed that pre-pubertal children with obesity and with NAFLD had lower z-scores of circulating leptin levels than children without NAFLD. Furthermore, children with low z-scores of circulating leptin levels had more often a severe degree of steatosis hepatis than children with normal z-scores of circulating leptin levels. In our study cohort, pre-pubertal children with obesity and with low z-scores of circulating leptin levels are characterized by an altered metabolic profile including higher triglyceride, insulin and c-peptide concentrations compared to children with normal z-scores of circulating leptin levels. We hypothesize that the group of pre-pubertal children with obesity and with low z-scores of circulating leptin levels could possibly benefit from treatment with Metreleptin in terms of an improvement in parameters of liver metabolism and potentially also weight loss. Further research is needed, to better characterize the phenotype of relative leptin deficiency in the context of obesity in childhood, clarify the underlying pathophysiology, and understand potential implications for designing targeted therapeutic interventions. One crucial step in this direction will be the development of updated and extended reference values for circulating leptin levels during childhood and adolescence including cohorts of children and adolescents with obesity and with extreme obesity.

## Data Availability Statement

The original contributions presented in the study are included in the article/supplementary material. Further inquiries can be directed to the corresponding author.

## Ethics Statement

The institutional ethical review board of the University of Ulm approved all study proceedings. Written informed consent to participate in this study was provided by the participants’ legal guardian/next of kin.

## Author Contributions

SB and MW researched data and wrote the manuscript. WK collected patient’s data, reviewed and edited the manuscript. CD and JvS reviewed and edited the manuscript. All authors had final approval of the submitted and published versions.

## Funding

This study received funding from FERRING GmbH. The funder was not involved in the study design, collection, analysis, interpretation of data, the writing of this article or the decision to submit it for publication. All authors declare no other competing interests.

## Conflict of Interest

The authors declare that the research was conducted in the absence of any commercial or financial relationships that could be construed as a potential conflict of interest.

## Publisher’s Note

All claims expressed in this article are solely those of the authors and do not necessarily represent those of their affiliated organizations, or those of the publisher, the editors and the reviewers. Any product that may be evaluated in this article, or claim that may be made by its manufacturer, is not guaranteed or endorsed by the publisher.
